# Psychosocial Experiences, Challenges, and Recommendations for Care Delivery among Partners of Breast Cancer Survivors: A Qualitative Study

**DOI:** 10.3390/ijerph20042786

**Published:** 2023-02-04

**Authors:** Chiara Acquati, Katharine J. Head, Kevin L. Rand, Jennifer S. Alwine, Danielle Nicole Short, Andrea A. Cohee, Victoria L. Champion, Claire Burke Draucker

**Affiliations:** 1 Graduate College of Social Work, University of Houston, Houston, TX 77004, USA; 2 Tilman J. Fertitta Family College of Medicine, University of Houston, Houston, TX 77004, USA; 3 Department of Health Disparities Research, The University of Texas MD Anderson Cancer Center, Houston, TX 77030, USA; 4 Department of Communication Studies, School of Liberal Arts, Indiana University-Purdue University Indianapolis, Indianapolis, IN 46202, USA; 5 School of Science, Indiana University-Purdue University Indianapolis, Indianapolis, IN 46202, USA; 6 School of Nursing, Indiana University, Indianapolis, IN 46202, USA; 7 Melvin and Bren Simon Comprehensive Cancer Center, Indiana University, Indianapolis, IN 46202, USA

**Keywords:** partners, caregiving, breast cancer, coping, psychosocial oncology, qualitative research

## Abstract

For women diagnosed with breast cancer, partners are consistently identified as the primary support person. Despite growing consensus about the psychosocial experience and unmet needs of cancer caregivers, limited evidence exists about strategies to offer partner-centered care across the cancer continuum. This study describes challenges endured by partners of breast cancer survivors (BCS), strategies implemented to manage these experiences, and recommendations for healthcare providers to inform targeted psychosocial care. Using convenience sampling, 22 partners of female BCS were recruited and completed semi-structured interviews. Conventional content analysis was used to code and synthesize findings. Participants described undergoing five experiences in their role as romantic partners: (a) assuming the role of caregiver, (b) becoming healthcare advocates for BCS, (c) connecting emotionally with the partner, (d) managing their own painful emotions, and (e) connecting with others for support. Experience-specific coping strategies and recommendations were identified. Romantic partners face multiple transitions across the cancer care continuum, which warrant investigation to sustain their well-being and active participation in illness management. Psychosocial interventions for this group will benefit from flexible implementation and attention to care delivery, mental health, and supportive/social needs.

## 1. Introduction

The societal value of caregiving for persons with cancer is of increasing importance due to aging demographics, newly emerging therapeutic approaches, and increased utilization of outpatient and home-based care delivery models [1,2,3,4,5]. Caregivers assist with care coordination, symptom management, and they provide emotional support, while also facing negative physical, psychosocial, and financial consequences [4,5,6,7,8,9,10]. Psychosocial oncology researchers have identified key priorities for assisting caregivers, including the understanding of the burdens associated with this role, developing interventions to address their concerns, integrating caregiving in healthcare settings, and providing technological resources [1,2,7,11]. An international Delphi survey conducted by Lambert and collaborators [1] highlighted the importance of eliciting caregivers’ perspectives for the optimal provision of supportive/information resources and the necessity for sustainable and caregiver-driven intervention approaches [2].

For women diagnosed with breast cancer, the spouse, partner, or significant other (hereafter referred to as partner) is consistently identified as the primary support person [12,13]. These couples are confronted with changes to their dyadic roles as well as disruptions to their communication, intimacy/sexuality, and relationship quality [14,15,16,17,18,19,20,21]. Approximately 62% of partners of cancer survivors experience clinically significant emotional distress [16]. Moreover, they report poorer quality of life and well-being than other caregiver populations [22,23,24,25]. Partners of BCS indicate more quality of life problems than partners of healthy women up to 14 years after diagnosis and treatment [26], are at increased risk of being hospitalized with an affective disorder [27], and are more likely to use antidepressant medication [28].

Studies of partners of BCS contribute to greater understanding of their psychosocial experiences [29,30,31,32]. A systematic review [29] revealed that they are significantly affected by the patients’ illness, rely on internal resources and the support of others to cope, and want healthcare systems to provide them with information needed to support their partners/wives [29,33]. Male partners can feel overwhelmed and ill-prepared to negotiate medical care for the BCS, cope with their own psychosocial needs and those of the BCS, manage financial concerns, and maintain daily activities [32,33,34]. They often use avoidant coping or protective buffering to safeguard the BCS’s well-being, although these approaches may lead to a sense of exclusion and reduced self-expression [30,35,36,37].

Despite growing consensus that healthcare providers should provide support for partners of BCS [1,2], limited evidence exists about best practices to offer partner-centered interventions across the continuum of care. Moreover, while many qualitative studies have provided in-depth information about partners’ experiences, few have comprehensively addressed the challenges, coping strategies, and professional support needs of partners. Learning about these issues from the perspective of the partners themselves is key to designing interventions to improve partners’ outcomes in the context of breast cancer survivorship. Therefore, the purpose of this study is to identify the challenges partners of BCS encounter, the strategies used to cope with these challenges, and the recommendations they have for providers.

## 2. Materials and Methods

Qualitative description, as outlined by Sandelowski [38], guided the present work. Qualitative description is a method used to produce a comprehensive and straightforward summary of stakeholders’ accounts about a specific healthcare experience. Purposive sampling, semi-structured interviews, and standard content analysis are common in qualitative description [38]. The study is reported in accordance with the Consolidated Criteria for Reporting Qualitative Studies (COREQ) guidelines (see the Supplementary Materials).

### 2.1. Participants

Potential participants included persons aged 18 years and older who self-identified as a current partner of a person diagnosed with non-metastatic breast cancer (stages 0–III) at any time in the past. Both partners and BCS could be any biological sex or have any gender identity. Partners were not expected to have been in a committed relationship with the BCS at time of diagnosis, just at the time of interview. Thus, those who were not in a relationship at study enrollment were considered ineligible. In alignment with infection prevention protocols that were required at the time due to the pandemic, interviews were conducted by phone or teleconferencing platform. Therefore, inclusion criteria required having access to a telephone or the internet. Finally, partners had to be English-speaking and live in the United States.

### 2.2. Recruitment and Enrollment

The study was approved by the Institutional Review Board of Indiana University (#1905795920). Participants were recruited using three recruitment strategies: (1) paid advertisements on social media (Facebook), (2) a statewide research participant registry, and (3) word-of-mouth/snowball sampling. Facebook ads ran daily for four weeks and targeted adults who lived within 50 miles of the study site and who had “liked” at least one local or national breast cancer organization. The statewide research participant registry is composed of over 22,000 volunteers interested in health sciences research. People who, upon enrollment in the registry, indicated a personal experience with breast cancer (their own or a partner’s) and expressed interest in breast cancer trials were emailed a study information letter with a link to the online study portal. Potential participants were also recruited through word-of-mouth using professional networks at a comprehensive cancer center. These networks included breast oncologists, community engagement specialists, nurses, psychologists, and faculty members. Additional efforts were made to recruit partners who identified as Black/African American, a group that is historically underrepresented in the partner-focused psychosocial oncology research [39,40,41].

Interested persons were directed to an online portal where they completed a brief eligibility screening and were provided a study information sheet. Those who completed the survey and were eligible were invited to provide sociodemographic information. Partners were also queried about the time since diagnosis, treatment completion, and recurrence. A research assistant called or emailed eligible individuals on the basis of their preferred contact method and provided additional, scripted information about the study. If the person remained interested, an individual interview was scheduled. A waiver of written informed consent had been obtained, and all participants agreed verbally to participate. Because potential participants worked and had multiple personal obligations, members of the research team contacted each eligible person up to four times at various times and days of the week to enhance enrollment.

Forty-three eligible persons responded to the recruitment survey and provided their contact information. Twenty-five (58%) were reached within four attempts and scheduled for an interview. This response rate is similar to other studies of BCS partners, caregivers, or dyads [16,17,42]. Two individuals were ineligible as they were not currently in a committed relationship with the BCS, and twenty-three (92% of those scheduled for an interview) completed the interview. Despite enrollment being open to partners of people from any sex or gender identity, all were partners of female BCS. There was a female partner who completed the interview and whose information was not combined with the rest of the sample out of concern that the findings may not be representative.

### 2.3. Data Collection

A female member of the research team (A.A.C.) conducted the interviews using a semi-structured interview guide, which was developed by an interdisciplinary team of behavioral oncology researchers and qualitative experts. Questions focused on self-reported challenges experienced during and following treatment and strategies implemented to cope with them. Participants were also invited to provide recommendations for how healthcare providers might best support partners of BCS. If a participant was not in a relationship with the BCS at time of diagnosis, questions were adapted to focus on the post-treatment phase only. A USD 25 gift-card was provided to compensate participants for their time. The research team determined the incentive to be in line with other qualitative studies conducted in this population [43,44,45]. All interviews were audio-recorded and transcribed for analysis. Two members of the research team (A.A.C. and J.S.A.) checked transcripts for accuracy. To protect confidentiality, any identifying information was removed, and a unique identifier was assigned to each participant.

### 2.4. Data Analysis

A standard content analysis approach [46] was used to analyze the participants’ narratives. The analytic team included investigators with expertise in cancer survivorship, partnered relationships (A.A.C.), and qualitative methodology (C.B.D.), in addition to a nursing graduate student (J.S.A.). Data analysis occurred in six steps. First, team members constructed a data display table, in which the main topics (e.g., challenges, strategies, suggestions for providers) were used as column headers and each row was labeled with the participant identification numbers. Second, after reading the transcripts in their entirety, a code was assigned to each remark related to the study aims. Third, final codes were placed in the appropriate cells in the data display table (e.g., participant PA-001 X *Needing information/knowledge about cancer*). Through discussion and consensus, the analytic team grouped similar codes within each column into categories. Then, the categories were organized into five groups (A.A.C. and C.B.D.) representing common psychosocial experiences of the participants. Narrative summaries of challenges, strategies, and recommendations associated with each experience were compiled. Finally, summaries were presented to the investigative team, discussed, and finalized.

## 3. Results

The sample includes 22 male partners of female BCS (mean age 57.5 years, SD = 10.4, range 37–80 years). Table 1 presents relevant sociodemographic information. Participants identified five experiences they underwent as a romantic partner. These included (a) becoming the primary caregiver for the BCS, (b) becoming healthcare advocates, (c) connecting emotionally with the BCS, (d) managing their own painful emotions, and (e) connecting with others for support. For each experience, participants identified challenges they encountered, strategies used to address them, as well as related recommendations for healthcare providers. Findings are presented in Table 2.

### 3.1. Experience I: Becoming Caregivers for the BCS

Twenty (87%) participants described becoming caregivers for the BCS due to the survivors’ multiple care needs, especially after surgeries and treatments. One participant stressed that he had to “*become a nurse overnight*”. Many managed the BCS’s medications, took care of their drains, and accompanied them to the bathroom. One participant described how he set an alarm for rotating his wife’s Tylenol^®^ and pain pills every two hours for two weeks. In addition to providing personal care for the BCS, many participants were also responsible for day-to-day household tasks. After the period of initial recovery, participants continued with caregiving activities, many of which were aimed at reducing cancer recurrence. Participants bought and prepared healthy foods; urged the BCS to exercise or, if needed, to rest; and encouraged them to continue their medication regime of aromatase inhibitors despite side effects.

*Challenges.* Many challenges were reported by participants in their role as caregivers. Overall, they felt they did not possess the skills needed to provide medical care, such as managing medications and tending to surgical drains. Some acknowledged that caregiving could be all consuming, and several needed to take time off from work or use Family Medical Leave Act (FMLA) benefits. Family and friends often offered to help with day-to-day tasks, but at times participants found it difficult to “*coordinate volunteers*”. One common challenge was that caregiving caused friction in the couples’ relationship. After treatment, some participants were frustrated that the BCS did not take their advice about healthy behaviors but recognized survivors were also frustrated with having their health-related choices and behaviors second-guessed.

*Strategies.* Participants managed their caregiving responsibilities in a variety of ways. Many called on their own resilience and went into “*battle mode*” by not “*letting the ‘c’ word [cancer] rule*”. Some learned to relate to the BCS in new ways when providing care. For example, one participant described how he learned to ask his wife what she needed and to negotiate when he should “*step in*”.

*Recommendations for Providers*. While most participants acclimated to caregiving responsibilities, several would have liked healthcare providers to offer additional training on tasks such as handling medication schedules, checking drainage tubes and bags, managing the BCS’s diet, and finding ways to keep the BCS comfortable.

### 3.2. Experience II: Becoming Healthcare Advocates for the BCS

Nineteen (83%) participants indicated that, in addition to their role as primary caregiver, they simultaneously became a healthcare “*advocate*” for BCS. Many felt responsible for absorbing, interpreting, and keeping track of medical information for the BCS and wanted to be an active participant in making decisions.

*Challenges.* Participants experienced challenges while advocating for the BCS, the most salient of which was a lack of knowledge about breast cancer and limited skills in advocating for better or different care. Many felt unprepared to take on this role as they were unfamiliar with the short- and long-term effects of breast cancer treatments. Some struggled to understand the BCS’s fatigue, “*chemo brain*” (i.e., cancer-related cognitive impairment), mood swings, pain, and neuropathy. Participants also had trouble navigating varying treatment plans recommended by multiple providers, interpreting complicated test results, responding to treatment decisions that were often made rapidly, and understanding new treatment technologies. Moreover, some participants felt excluded from the BCS’s healthcare. One participant stated that he felt like a “*secondary character in the story*”. Many commented on how this feeling was magnified during the COVID-19 pandemic. As partners were not allowed to enter cancer centers or to attend BCS’s appointments in person because of hospital restrictions, they felt ignored during visits even when joining via FaceTime.

*Strategies.* Participants used multiple strategies to advocate for the BCS. Most took the BCS to medical appointments and treatments and regularly communicated with healthcare providers about their partner’s diagnosis, treatment plan, and prognosis. Many participants came to feel comfortable talking with the BCS’s doctors, though several needed help “*deciphering the lingo*”. If they did not get the information they needed from providers, they tried to find quality information from other sources (e.g., online forums).

*Recommendations for Providers*. Participants stated that providers should develop tools to help partners understand medical information and provide education tailored to the BCS’s stage of cancer and individual treatment plan. Several expressed wanting instant access to information on a website or a “*live chat with a trained person*”. Some appreciated working with navigators who guided the couple through the cancer journey.

### 3.3. Experience III: Connecting Emotionally with the BCS

Sixteen (70%) participants described their attempts to connect emotionally with the BCS. Most tried to be reassuring, supportive, sensitive, and compassionate. They wanted to “*be there for*” the BCS and to provide them with comfort, hope, and encouragement.

*Challenges.* Several participants felt disconnected from the BCS due to disruptions in communication. Some reported they did not know the “*right things to say*”, and others wanted to encourage the BCS to talk about what they were experiencing but feared being “*too pushy*”. Participants were hesitant to sound overly optimistic, feared of discounting BCS’s feelings, or were unsure about giving advice. Many participants were struggling with their own feelings, especially fear of losing their partners or concerns over a cancer recurrence but felt reluctant to share these feelings to avoid burdening the BCS. One participant was especially hesitant to share his sadness with his wife and thus felt distant from her. Some indicated that communication problems caused resentment and took a “*toll*” on the relationship, with one of our participants stating that he was unsure if the relationship would recover.

*Strategies.* Despite these challenges, most participants found ways to maintain a connection with the BCS. They expressed their closeness by showing care, listening rather than arguing, trying not to “*fix things*”, and spending time “*just talking*”. They reassured the BCS that they would *“be there”,* that the BCS was not alone, and the couple would “*pull together as a team*”. Some participants learned to give the BCS time and space to express their feelings and needs.

*Recommendations for Providers*. A few participants suggested that a couple counselor or therapist who “*knew something about cancer*” and could serve as an “*anchor*” for the couple should be made available. Participants indicated that such a professional could relieve some of the emotional burden they also experienced firsthand.

### 3.4. Experience IV: Managing Painful Feelings

Twenty (87%) participants described managing their own painful emotions. In response to BCS’s cancer, most participants experienced intense and negative emotions such as fear, anxiety, and worry. One participant said he experienced “*a black cloud of fear*”. These were due to concerns that cancer would spread or re-occur, or that the BC survivor would die. Some participants also experienced surprise and shock at the diagnosis and grief and depression throughout the course of treatment. Several felt helpless because the cancer was “*out of my hands*”. One participant described the pain of having to watch the “*poison*” of chemotherapy go into his wife’s body. Others were distressed by having to endure watching “*someone you love so much going through the pain and fear*” without being able to protect them.

*Challenges*. Participants experienced several challenges managing their painful feelings, especially their fear, and often “*bottled up*” their emotions. Some felt it was not acceptable to express their feelings. One participant stated, “*Men have to emotionally suck it up and go with it*”. Others kept their sentiments to themselves to avoid burdening the survivor.

*Strategies.* Despite these challenges, some participants wanted to be able to “*to feel the full range of emotions*”. Others sought outlets to get a break from their feelings by taking trips, playing golf, running, journaling, and spending time with friends as a reprieve from their worries.

*Recommendations for Providers*. Some participants recommended that professional counselors could be of help to partners who were struggling with their own painful feelings/emotions.

### 3.5. Experience V: Connecting with Others for Support

Eighteen (78%) participants described connecting with others for emotional and instrumental support. Many talked with others who understood what they were going through and to whom they could vent their feelings and express concerns. In some instances, participants were particularly interested in connecting with other men whose partners had had breast cancer. Some partners sought out a confidant whom they knew and trusted, whereas others felt strangers were most helpful. Conversely, few participants indicated that they did not desire to share their feelings or get advice from others at all. One stated he wanted others to “*stay away*”.

*Challenges.* Some participants experienced difficulties in connecting with others for support. A few felt they had no one to offer the kind of assistance they needed. One stated he did not have many friends, and there was no one with whom to “*grab lunch*”. Others were reluctant to “*share their stories*” because they were “*private people*” and thus did not wish to reveal personal details about their lives. These participants kept their feelings to themselves and were “*closed off*” from others. Some indicated that the challenge of obtaining support from others during the breast cancer journey was related to gender-based expectations because “*men do not talk*”.

*Strategies.* Many participants shared they had indeed connected with others from whom they received support. They “*vented*” to family members, friends, clergy, and church members and found this helped them “*decompress*”. One participant described the comfort he received from having a close friend he could reach out to but “*doesn’t have to*”, and another described asking for prayers. Participants sought advice about how to respond to the BCS’s feelings, how to “*handle all of this*”, or how to prepare for “*what’s going to happen*”. One participant stated that he wanted somebody to be there who could understand the “*man’s side of it*”. Another participant indicated that he was able to express fears he was trying to “*suppress*” or hide from his wife with another man whose wife had breast cancer. Others had interactions with men or BCS who had “*been through it*” and who could thus provide informational support in the form of “*firsthand*” guidance.

*Recommendations for Providers.* A few participants suggested that counseling could help partners “*open up about what is going on*”. Several recommended cancer support groups for partners. They stated that these groups had helped them build networks, exposed them to a “*range of experiences*”, introduced them to people with similar caregiving experiences, and provided opportunities to share. Others recommended that healthcare providers should arrange for a “*third person*” mentor who was not in their “*inner circle*”, making it easier for participants to share personal information, needs, and feelings.

## 4. Discussion

Partners often become the primary caregivers of women with breast cancer, a role that is associated with significant impact on their well-being [1,2,3,4,10,14,18,26,30,37,47]. With increasing attention to the inclusion of caregivers for optimizing interventions and outcomes, it is critical to understand the transitions partners experience across the continuum of care and their preferences for psychosocial care delivery. The present study qualitatively captured their challenges, coping strategies, and recommendations for providers. Findings centered on five themes including (a) assuming the role of caregivers, (b) becoming healthcare advocates, (c) connecting emotionally with their partners, (d) managing their own painful emotions, and (e) connecting with others for support.

While the consequences of caregiving are well documented [9,10,11], a salient finding from this study was the profound impact of assuming the role of caregiver and healthcare advocate on the identity of male partners of BCS. Data highlight the anguish associated with their dual role and how their distress was exacerbated by the feeling of being unprepared to complete direct medical tasks, managing symptoms, administering medications, and engaging in treatment decisions. Although the involvement of informal caregivers and partners in decision making and illness management is associated with increased satisfaction and adherence to treatments [48], the growing tendency for home care approaches introduces new challenges and tasks for partners.

The emotional distress described by the participants, especially fear of losing the BCS and depression, resonated with prior studies that revealed how both BCS and partners report high levels of distress [47,49]. In fact, studies have indicated that between 20 and 40% of partners experience sustained, clinically significant depressive symptoms years after treatment has ended [28,47,50]. It is also important to note that this study was conducted during the COVID-19 pandemic and that current findings may have been influenced by social distancing measures. These protocols have restricted access to formal and informal support resources, with documented increase in the intensity and burden of caregiving tasks [51,52]. Cancer caregivers have also faced additional stressors while navigating considerable physical and psychosocial demands resulting from increased financial concerns, lack of access to family members, and social networks [51,53,54,55].

Findings from this study also highlight gender-based norms in relation to coping strategies and response to the role of caregiver [35,36]. Several participants indicated it was not “*macho*” to express their feelings. This datum is consistent with prior research that revealed how male partners of female BCS may face high levels of vulnerability because of their inability to disclose their feelings, a challenge often associated with stereotypical representations of masculinity [21,29,56,57]. Similarly, partners tended to utilize protective buffering as a strategy to preserve the well-being of the cancer survivor despite the documented negative consequences on individual and dyadic outcomes [30,35,36,37]. For example, Perndorfer et al. [17] demonstrated how protective buffering contributed to greater fear of recurrence and decreased intimacy among patients and partners. Finally, participants described challenges connecting emotionally with survivors and the desire to receive counselling and support renegotiating closeness and intimacy [26,58,59]. If we consider cancer as a “we-disease” [60], it follows that couples adapt to it as an emotional system [61], and each partner mutually influences distress, coping behaviors, and communication exchanges in a way that must be accounted for when designing interventions [19,62,63,64,65].

### 4.1. Implications for Psychosocial Oncology

Several programs have been developed for caregivers of cancer survivors to improve their quality of life, symptoms, and physical health [40,41,42]. However, limited attention has been dedicated to ground these approaches in partners’ expressed needs and preferences for care [1,2,7,11]. Our participants felt that healthcare providers could and should help them deal with the challenges they faced. They underscored how interventions should target four key domains: (1) enhance partners’ ability to provide direct care and skills needed to interact with and navigate the healthcare system, (2) sustain the dyad’s ability to connect emotionally and nurture their bond, (3) develop stress management and coping skills, and (4) foster a sense of connection with others. Interventions for partners of cancer survivors have traditionally been classified in psychoeducation, skills training, and counseling [42,43]. However, present findings underscore how an integrated approach may be beneficial. On the basis of these suggestions, we recommend that providers offer practical training on managing complex personal care tasks, such as tending to drain and managing medications. Moreover, providers should provide easily accessible and clear information in the form of “tools” about cancer across the continuum of care, understanding results of diagnostic tests and procedures, and evaluating risks and benefits of available treatments. These tools can be supplemented with live chats or interactive websites. Oncology social workers and the multidisciplinary healthcare team play an important function in helping couples navigate the cancer experience [66]. We propose that therapists who specialize in cancer survivorship and couple counselors with expertise in illness-related stressors be accessible to partners at all stages of the cancer trajectory. Support groups, peer-mentors, and cancer websites are ancillary strategies that providers should make easily accessible to partners.

Our findings also pointed to considerations pertaining to the preferred format and delivery of future interventions for partners [2,11]. *E*-health or *m*-health approaches were considered suitable to gain education and increase self-efficacy related to breast cancer care and treatments, while counselors and support groups were requested to address couples’ concerns and foster connections with others respectively. Furthermore, our findings suggest that interventions to assist partners of female BCS should be comprehensive, targeted, and accessible. Given recent advances in technology-based approaches, these programs can offer greater accessibility, be cost-effective, and can be delivered in a self-paced and tailored manner [67,68]. It is, however, important to note that as participants’ needs varied, referrals to interventions would need to be tailored to their unique experience and preferences [42,43].

### 4.2. Study Strengths and Limitations

Participants were offered an opportunity to freely share their experiences, thus providing rich details about the psychosocial challenges partners face. Additionally, the transition to online interviews eliminated the need for participants to travel to an interview site, offered greater flexibility in scheduling, and provided a greater sense of anonymity for some. These findings should, however, be considered in the context of certain study limitations. Conducting interviews via teleconferencing may have dissuaded those unfamiliar with the technology or without ongoing access to internet services from participating. In addition, the study used a cross-sectional design, and causal inferences about the influence of the reported challenges/strategies on the partners’ well-being could not be determined. Similarly, it was not possible to capture nuanced changes across the care continuum. Moreover, variations between time of diagnosis and the interview could have introduced some memory, cohort, or history effects. The sample included only male persons in heterosexual relationships (*n* = 22), thus limiting generalizability of the present findings to female partners or persons in lesbian, gay, bisexual, transgender, or queer relationships. Despite efforts to recruit a diverse sample, the study lacked Hispanic/Latino (*n* = 0) and Asian (*n* = 1) representation. Future research should include understudied and underserved groups of partners; utilize longitudinal designs; and examine associations between partners and survivors’ experiences, well-being, and healthcare experiences.

## 5. Conclusions

Partners face multiple transitions across the cancer care continuum, which warrant investigation to sustain their well-being and active participation in illness management. Findings from this qualitative work underscore the change in personal identity and role partners endure when becoming caregivers and advocates, as well as challenges that characterize the relationship with the BCS, the larger social network, and attempts to cope effectively with their own emotions. This contribution therefore provides additional evidence of the psychosocial, emotional, and relational challenges experienced by partners during survivorship. A noteworthy finding is that partners are well-poised to articulate their needs and preferences for care from healthcare providers, and their voices should be considered when therapeutic strategies are developed. Psychosocial interventions for this group will benefit from flexible implementation and attention to educational needs regarding direct care delivery, involvement in care coordination, stress management skills, and communication strategies that sustain the relationship with the care-recipient. In addition, mental health issues and social isolation reported by partners suggest that future work must also privilege these domains of care.

## Figures and Tables

**Table 1:** Demographic characteristics of the participants (*n* = 22).

Variables	Frequency	Percentage (%)
***Age***		
30–39	1	4.5
40–49	4	18.2
50–59	7	31.8
60–69	7	31.8
70–79	1	4.5
80+	1	4.5
Not reported	1	4.5
***Race***		
Black/African American	12	54.5
White/Caucasian	9	40.9
Asian/Asian American	1	4.5
***Education***		
High school graduate	2	9.1
Some college/certification courses	3	13.6
College graduate	10	45.5
Graduate/professional degree	6	27.3
Not reported	1	4.5
***Ability to Pay for Basic Goods***		
No difficulty	20	90.9
Some difficulty	1	4.5
A lot of difficulty	-	-
Not reported	1	4.5
***Time Since BC Survivors’ Diagnosis***		
≤1 year	3	13.6
2–5 years	5	22.7
6–10 years	10	45.5
>10 years	2	9.1
Not reported	2	9.1

**Table 2:** Overview of partners’ self-reported experiences, challenges, and coping strategies. Recommendations for providers are included with exemplar quotes.

Experiences	Challenges	Coping Strategies	Recommendations for Healthcare Providers	Quotes
Becoming Caregivers for the Breast Cancer Survivor (BCS)	Lack of skills needed to provide personal medical care; Caregiving tasks/role interfering with the couple relationship	Going into “battle mode”; Relating to the BCS in new ways	Provide education or training on medical care tasks (e.g., medication schedules)	*Because you know, you would hear her moan or something and like you had to rotate the Tylenol with the pain pills every two hours, three hours. So, you would sleep a little bit and then the alarm would go off. And so I had to get up, get the pills and the alarm would go off three hours later again. Because it was like every three hours you would switch to the pill. And that went on around the clock 24 h.* PA-015 *I try to have a role, but it’s got to be her choice. So, if she wants to do it and she asks me to help, then I will, but if she stops. I say okay. I’ll let you do your thing until you need me again.* PA-011
Becoming Healthcare Advocates for the BCS	Not having cancer-specific knowledge; Not knowing how to advocate for better care; Feeling left out of BCS care (exacerbated by COVID-19 pandemic)	Learning to communicate with healthcare providers; Locating/accessing medical information from other sources	Develop tools to help partners understand medical information; Provide websites/live chats with a trained person to offer accessible cancer-related information; Provide nurse navigators	*Just having another set of eyes and ears and being able to absorb the information that I think her in the role as a patient. And particularly when you’re hearing pretty intense news, it’s really hard to absorb that. I think that was another thing was just being able to go and listen and participate and serve as sort of a collective memory and being able to help keep track of some of the details.* PA-04 *I would always ask them ‘What does that mean really to a regular person?’ And they would always break things down.… I was able to decipher the lingo that [the plastic surgeon] was using. I’m like okay, I got you.* PA-024
Connecting Emotionally with the BCS	Not knowing what to say; Not wanting to share their own feelings; Experiencing communication disruptions with BCS	Listening instead of trying to fix things; Spending time talking; Reassuring the BCS that they were “there” for her/them; Viewing the dyad as a “team”	Provide couple counselors with cancer-specific knowledge	*I’m not sure I knew enough to say the right things, to be as supportive as I could have been. It wasn’t for lack of trying, it was just for lack of, just not knowing.* PA-01 *I try to comfort her. I just sit with her and we talk about it. I let her know that I’m still there and it’s something that we’re going through together. She’s not going by herself. I’m there to go through it with her as a couple and try to help her and encourage her that it’s going to still be all right.* PA-010
Managing Painful Feelings	Bottling up emotions; Needing to remain “macho”	Allowing themselves to feel the full range of feelings; Finding outlets to get a break from feelings	Provide professional counselors for partners	*Let me take a trip and go play golf. Let me go to homecoming and have fun with my fraternity brothers. As much as they need an outlet, we need an outlet to, or I’ll just say me. As much as she needed an outlet to cope and deal with things, I need an outlet to cope and deal with things too.* PA-013 *You can’t help but think the worst, no matter how much you go this is going to be okay, and you’re going to be one of the ones that makes it, and you’ve been strong, you’ve been healthy and you’ve got good care and all those things. You can say that all you want, and in those quiet moments, you’re like yeah, but it might not go that way, because sometimes it doesn’t, and even now it doesn’t matter how well things seem to be going, you can’t help but have that tiny little black cloud over your head going what if when we go to the doctor this time, he sees something?* PA-003
Connecting with Others for Support	No or few available support persons; Reluctance to share negative emotions; Expression of feelings constrained by gender norms	Venting to trusted others to decompress; Finding others who understood their situation; Seeking advice from others including other partners of BCS	Provide access to professional counselors; Provide access to support groups; Connect with “third person” mentors; Develop cancer websites or Facebook groups	*I just know that at least for me, it would have been nice to have someone that got it. I didn’t not necessarily even have a lived experience, but just understands. And didn’t feel like a therapist who was constantly going through an assessment just to be able to have an open conversation about it.* PA-016 *The [friends and family] that I trusted to zip their lips, I would definitely say I expressed my thoughts and concerns and my insecurities about the situation… My insecurity was the thought that I could lose her.* PA-014

## Data Availability

Data analyzed in the current study are available from the corresponding authors on reasonable request.

## Supplementary Materials

The following supporting information can be downloaded at https://www.mdpi.com/article/10.3390/ijerph20042786/s1. Consolidated Criteria for Reporting Qualitative Studies (COREQ).
